# Risks for HIV and other sexually transmitted infections among Asian men who have sex with men in Vancouver, British Columbia: a cross-sectional survey

**DOI:** 10.1186/1471-2458-13-763

**Published:** 2013-08-16

**Authors:** Thiha Maung Maung, Becky Chen, David M Moore, Keith Chan, Steve Kanters, Warren Michelow, Robert S Hogg, Nadine Nakamura, Wayne Robert, Reka Gustafson, Mark Gilbert

**Affiliations:** 1 Faculty of Health Sciences, Simon Fraser University, Burnaby, BC, Canada; 2 Faculty of Medicine, University of British Columbia, Vancouver, BC, Canada; 3 British Columbia Centre for Excellence in HIV/AIDS, Vancouver, BC, Canada; 4 Health Initiative for Men, Vancouver, BC, Canada; 5 Vancouver, Coastal Health, Vancouver, BC, Canada; 6 Division of STI/HIV Prevention and Control, British Columbia Centre for Disease Control, Vancouver, BC, Canada; 7 University of La Verne, La Verne, CA, USA

**Keywords:** HIV, Homosexuality, Men who have sex with men, Asian

## Abstract

**Background:**

Individuals of Asian heritage represent the largest ethnic minority in Canada. Approximately 10% of the new HIV diagnoses in men in British Columbia occur among Asian-Canadians. However, the HIV risk patterns of Asian men who have sex with men (MSM) have not been extensively studied.

**Methods:**

Participants aged ≥ 19 years were enrolled in a venue-based HIV serobehavioural survey of MSM in Vancouver, Canada. We compared the demographic characteristics, risk behaviours, and prevalence of HIV and other sexual and blood borne infections between Asian and non-Asian MSM using bivariate analysis and logistic regression confounder modelling.

**Results:**

Amongst 1132 participants, 110 (9.7%) self-identified as Asian. Asian participants were younger than non-Asian participants (median age 29 vs. 32 years; p < 0.001), but otherwise did not differ from other study participants. HIV prevalence was lower among Asian MSM compared to Non-Asian MSM (3.7% vs 19.0%, p <0.001). Among men who self-reported as HIV negative or unknown we found no differences in unprotected anal intercourse (UAI) with a discordant or unknown serostatus partner in the previous six months (11 vs. 13%; p = 0.503). However, Asian MSM were less likely to report ever using injection drugs (10.8% vs. 19.2%; p = 0.043) or using alcohol before having sex (52% vs. 64.4%; p = 0.017).

**Conclusions:**

Asian MSM in our study reported similar rates of UAI as non-Asian MSM, but had a lower prevalence of HIV infection. Other factors, such as the use of drugs and alcohol, in relation to sex, may partly explain these differences. However this requires further investigation.

## Background

Men who have sex with men (MSM) in Canada continue to be heavily affected by HIV and sexually transmitted infections (STI), accounting for 46.6% of the total new HIV infections in 2011 [1]. In the United States, ethnic minorities, predominantly black and Latino men, are overly represented in the HIV epidemic among MSM [2]. However, relatively little has been written about ethnicity for MSM outside of the United States. Individuals whose family origins are in Asia represent the largest racial minority in Canada, making up 11% of the population [3]. However, 60% of new immigrants to Canada from 2001–2006 were from Asian countries. Furthermore, in major metropolitan cities, such as Toronto and Vancouver, individuals of Asian ancestry represent 30% and 40%, respectively, of the population [3].

In British Columbia, Asian-Canadians accounted for 11.9% of new HIV diagnoses in men in 2009, which is the largest number of any ethnic minority [4]. The majority of these infections are due to male-to-male transmission (M. Gilbert, personal communication). Despite the prominence and growth of the Asian-Canadian population, very little is known about HIV risk behaviours among Asian-Canadian MSM. Only a handful of studies have been conducted on HIV risk among Asian-Canadians [5-9].

In order to improve our understanding of Asian MSM and HIV risk behaviour we conducted a secondary analysis of a cross-sectional serobehavioural survey of MSM in Vancouver. We sought to evaluate the differences in demographic characteristics, risk behaviours and HIV prevalence among Asian and non-Asian MSM, and evaluate testing patterns for HIV and other STIs and blood borne infections among this population. In this analysis Asian MSM included participants who identified with any Asian ethnic or cultural group whether from east, west, central or south Asia.

## Methods

We used data from the ManCount Survey, the Vancouver site of M-Track, a behavioural and biological surveillance program for HIV and other sexually transmitted and blood-borne infections among MSM coordinated by the Public Health Agency of Canada. In Vancouver, the survey was conducted between August 2008 and February 2009. ManCount applied a venue-based, time-space sampling methodology [10] and recruited men 19 years of age and older who self-identified as an MSM at a selection of 20 community venues or events in Vancouver. Following informed consent, participants self-administered an English language survey and provided a blood sample on filter paper for a dried blood spot (DBS) specimen. The informed consent process, questionnaire administration and blood collection all took place on-site in the recruitment venues.

DBS specimens were tested for HIV and syphilis, at the National HIV Retrovirology Laboratories in Ottawa. The details of this testing is described elsewhere [11]. Ethics approval for the ManCount survey was obtained from Health Canada, Vancouver Coastal Health and the University of British Columbia. Ethics approval was also obtained from Simon Fraser University for this secondary data analysis.

For this study, Asian ethnicity was defined based on answers from two survey questions on ethnicity: “What are the ethnic or cultural origins of your ancestors?” (multiple responses permitted), and “Which single ethnic or cultural group do you most strongly identify with?” (single response permitted). Both questions provided a list of 26 examples of possible ethnicities followed by “etc.” to guide respondents. The list included Chinese, East Indian, Fillipino and Vietnamese as examples. Participants were identified as being of Asian ethnicity if any of the following criteria were met: i) reported as Asian ethnicity in response to the first question; or ii) reported solely as Asian ethnicities in response to the second question; or iii) those reported only as Asian ethnicities and “Canadian” in the same question. All other participants were classified as “non-Asian” for our analysis. We classified reported ethnicities as Asian according to Statistics Canada’s Ethnic Origin Reference Guide, 2006 Census [3].

We performed bivariate analyses comparing Asian and non-Asian participants with respect to demographic characteristics, HIV risk behaviour and HIV and other STI testing behaviour and results, using the Wilcoxon-Rank sum test and Fisher’s exact test. Unless otherwise stated, percentages are expressed from the total of respondents who answered a particular question or set of questions. We did not weight the analyses based on the site of recruitment. Both recent testing (within the past two years) and lifetime testing behaviours were considered. To remove, as best as possible, the effect of confounders on the observed association between ethnicity (Asian/non-Asian) and HIV/ST testing over the last two years, we developed confounder models using logistic regression analysis. We used a backward stepwise approach based on the magnitude of change in the coefficient of the explanatory variable of main interest. We stratified these models on the basis of age, using 35 years of age as the cut-off, since previously analyses from ManCount had found that testing behaviours differ between younger and older men [11], We used SAS version 9.1.3 (Cary, NC, USA) for all statistical analysis.

## Results

A total of 2805 individuals were approached for study participation, 1169 (41.7%) were enrolled and completed the survey and 1,132 (96%) provided a DBS sample suitable for testing and answered the ethnicity questions. Of these, the median age was 34 years (Interquartile range [IQR] 26–44 years), 87% (985/1129) resided in the metropolitan Vancouver area and 80% (902/1128) had at least some post-secondary education. A total of (935/1119) identified as being gay or queer, 10.8% (121) as being bisexual, 1.7% (19) as being straight and 2.9% (32) as being two-spirited and 1.1% (12) as having another sexual identity.

Of these, 9.7% (110/1132) of respondents self-identified as being of Asian ethnicity, 862 (76.2%) as having North-American or European ethnicity, 4.3% (49) as being Aboriginal and 9.8% (111) as having other ethnic identities. Of the Asian MSM, 60% (66) reported that they spoke English as a first language, while 40% (44) spoke the following languages as their first language: Cantonese (36.3%), Mandarin (13.6%), Tagalog (11.4%), Japanese (9.1%), Vietnamese (6.8%), Thai (4.6%), Punjabi (2.3%), Sinhala (2.3%), Spanish (2.3%), Korean (2.3%), Filipino (2.3%), French (2.3%) and Indonesian (2.3%).

Asian MSM were younger than other study participants (median age 29 years vs. 34 years; p < 0.001) and were more likely to have completed or had some post-secondary education (88.2% vs. 79.1%; p = 0.023), but did not differ in terms of income, sexual orientation or area of residence (Table 1). In terms of sexual behavior, a smaller proportion of Asian MSM reported >5 anal sex partners in their lifetime (43.6% vs. 58.5%; p = 0.006 for dichotomized variable) but we did not find any differences in the number of reported sex partners in the previous six months or having had any anal sex in the past six months (Table 2). We found that Asian MSM were less likely to report attending gay bars or nightclubs (52% vs. 64.4%; p = 0.017), but were just as likely to look for sex partners in bars or on the internet. Asian MSM were also less likely to report using alcohol before sex in the past six months (67.3% vs. 78.3%; p = 0.015) and were less likely to report ever having used injection drugs (10.8% vs. 19.2%; p = 0.043). However, we found no differences in the reported use of stimulants or psychedelics before sex. There were also no differences in the proportion of self-reported HIV negative participants who reported unprotected anal intercourse with a known HIV positive or unknown serostatus partner in the previous six months (our definition of risky sex). This was also true of men who self-reported as HIV positive. Asian MSM also appeared to judge their likelihood of acquiring HIV in their lifetime similarly to non-Asian MSM, with 39.8% reporting that it would be very unlikely (vs. 40.7% for non-Asian participants) and 40.9% reporting it unlikely (vs. 43.6% of non-Asians) that they would acquire HIV in their lifetime (p = 0.436).

**Table 1:** Demographic characteristics among Asian and non-Asian study participants in the ManCount Survey, Vancouver, Canada

**Characteristics**	**Non-Asian**	**Asian**	**p-value**
	**n = 1022 (%)**	**n = 110 (%)**	
Median age (IQR)	34 (26–44)	29 (25–38)	**<0.001**
Education			
Less than or completed high school	213/1018 (20.9)	13/110 (11.8)	**0.023**
Some or completed post-secondary	805/1018 (79.1)	97/110 (88.2)	
Income			
Under $30,000	388/998 (38.9)	50/108 (46.3)	0.30
$30,000 - $59,999	372/998 (37.3)	37/108 (34.3)	
$60,000 or more	238/998 (23.8)	21/108 (19.4)	
Sexual orientation			
Queer or gay	838/1010 (82.9)	97/109 (88.9)	0.23
Bisexual or two-spirit	142/1010 (14.1)	11/109 (10.1)	
Straight or other	30/1010 (2.9)	1/109 (0.9)	
Live in Metro Vancouver			
Yes	888/1019 (87.1)	97/110 (88.2)	0.88
No	131/1019(12.9)	13/110 (11.8)	
Recruitment venue			
Bar	557/1022 (54.5)	65/110 (59.1)	0.012
Bathhouse	37/1022 (3.6)	10/110 (9.1)	
Event	260/1022 (25.4)	24/110 (21.8)	
Community association	48/1022 (4.7)	6/110 (5.5)	
Business	120/1022 (11.7)	5/110 (4.5)	

**Table 2:** Bivariate comparison of HIV risk behaviours for Asian and non-Asian MSM

**Characteristics**	**Non-Asian**	**Asian**	**p-value**
	**n/N (%)**	**n/N (%)**	
Anal sex in past 6 months			
No	272/993 (27.4)	33/107 (30.8)	0.49
Yes	721/993 (72.6)	74/107 (69.2)	
Sex partners (oral or anal) in past 6 months			
None	83/994 (8.4)	7/108(6.5)	
Only one	229/994 (23)	29/108 (26.9)	0.89
2 to 5	349/994 (35.1)	36/108 (33.3)	
>5	333/994 (33.5)	36/108 (33.3)	
Lifetime number of anal sex partners			
None	64/906 (7.1)	9/101 (8.9)	
Only one	76/906 (8.4)	14/101 (13.9)	0.006
2 to 5	236/906 (26.0)	34/101 (33.7)	
>5	530/906 (58.5)	44/101 (43.5)	
Risky sex* in past six months by HIV negative or unknown serostatus participants	97/723 (13.4)	9/86 (10.5)	0.50
Risky sex**** in pasts six months among self-reported HIV + participants	46/180 (25.6)	2/3 (66.7)	0.17
Risky sex* in pasts six months in participants < 30 years of age	40/282(14.1)	4/42 (9.5)	0.41
Attend gay bars (at least once per month)	600/931 (64.4)	53/102 (52.0)	**0.017**
Attend bath houses (at least once per month)	103/924 (11.1)	13/101 (12.9)	0.619
Attend Pride Parade or Pride Festival	826/989 (83.5)	87/107 (81.3)	0.58
Search for sex partners on the internet	499/985 (50.7)	56/105 (53.3)	0.61
Search for sex partners in gay bars	552/1003 (55.0)	54/105 (51.4)	0.54
Received money, drugs, or goods/services in exchange for sex in past 6 months	131/963 (13.6)	13/104 (12.5)	0.88
History of injection drug use			
No	793/982 (80.8)	91/102 (89.2)	**0.043**
Yes	189/982 (19.2)	11/102 (10.8)	
Use of alcohol within 2 hours before sex the past 6 months			
No	215/989 (21.7)	35/107 (32.7)	**0.015**
Yes	774/989 (78.3)	72/107 (67.3)	
Use of stimulants or psychedelics** within 2 hours before or during sex in past 6 months			
No	720/975 (73.8)	84/104 (80.8)	0.15
Yes	255/975 (26.2)	20/104 (19.2)	
Perception of HIV infection in lifetime***			
Very unlikely	284/697 (40.7)	35/88 (39.8)	0.44
Unlikely	304/697 (43.6)	36/88 (40.9)	
Somewhat likely	95/697 (13.6)	13/88 (14.8)	
Likely	7/697 (1)	3/88 (3.4)	
Very Likely	7/697 (1)	1/88 (1.1)	

*Unprotected anal sex with HIV positive or unknown sero-status male partners (HIV negative or unknown only).

**ketamine, ecstasy, crystal meth, amphetamines, GHB or psychedelics.

***among self-reported HIV negative or unknown only.

****Unprotected anal sex with HIV negative or unknown sero-status male partners.

Table 3 compares the HIV and STI testing behaviors in Asian and non-Asian participants in our study. Asian participants were less likely to have ever been tested for HIV (75.0% vs. 86.8%; p = 0.004), but were similar to non-Asian men in terms of testing for HIV in the past two years (63.0% vs. 68.8%; p = 0.176). Asian MSM were less likely to be HIV positive, both by self-report (2.8% vs. 18.1%; p < 0.001), as well as by the DBS result taken during the survey (3.7% vs. 19.0%; p < 0.001). Of the 4 Asian participants who tested positive for HIV, 3 (75.0%) were aware of their infection, in comparison to 88.1% of HIV positive non-Asian participants. (p = 0.408) These differences in HIV prevalence were similar when the analysis was restricted to only those aged <30 years of age (1.8% vs. 7.2%) but did not reach statistical significance (p = 0.233). We also found lower proportions of ever testing for gonorrhea (p = 0.001) and hepatitis C (p < 0.001) among Asian participants, but no difference in ever testing for syphilis (p = 0.407). A lower proportion of Asian MSM reported testing for hepatitis C in the past two years (p = 0.22), but not for gonorrhea (p = 0.080) or syphilis (p = 0.407). Asian MSM were also less likely to have disclosed that they have male sex partners to a health care professional (70.4% vs. 80.6%; p = 0.016).

**Table 3:** Bivariate comparison of HIV sero-status and sexual and blood-borne infection testing experience for Asian and non-Asian MSM

**Characteristics**	**Non-Asian**	**Asian**	**p-value**
	**n/N (%)**	**n/N (%)**	
Ever been tested for HIV			
No	112/998 (11.2)	23/108 (21.3)	**0.004**
Yes	866/998 (86.8)	81/108 (75.0)	
Don’t know	20/998 (2.0)	4/108 (3.7)	
Tested for HIV in past 2 years*			
No	247/791 (31.2)	39/102 (38.2)	0.18
Yes	544/791 (68.8)	63/102 (61.8)	
Self-reported HIV serostatus			
Negative	746/1002 (74.5)	89/109 (81.7)	**<0.001**
Positive	181/1002 (18.1)	3/109 (2.8)	
Don’t know	75/1002 (7.5)	17/109 (15.6)	
HIV test result from dried blood sample			
Negative	807/996 (81.0)	104/108 (96.3)	**<0.001**
Positive	189/996 (19.0)	4/108 (3.7)	
HIV test result from dried blood sample in participants < 30 years of age			
Negative	335/361 (92.8)	54/55 (98.2)	0.23
Positive	26/361 (7.2)	1/55 (1.8)	
Disclosed male sex partners to a health care professional			
No	190/979 (19.4)	32/108 (29.6)	**0.016**
Yes	789/979 (80.6)	76/108 (70.4)	
Ever tested for gonorrhea			
No	213/978 (21.8)	39/108 (36.1)	**0.001**
Yes	688/978 (70.3)	58/108 (53.7)	
Don’t know	77/978 (7.9)	11/108 (10.2)	
Tested for gonorrhea in past 2 years			
No	448/954 (47)	59/105 (56.2)	0.080
Yes	506/954 (53)	46/105 (43.8)	
Ever tested for syphilis			
No	252/980 (25.7)	37/107 (34.6)	0.13
Yes	646/980 (65.9)	61/107 (57.0)	
Don’t know	82/980 (8.4)	9/107 (8.4)	
Tested for syphilis in past 2 years			
No	445/948 (46.9)	53/103 (51.5)	0.41
Yes	503/948 (53.1)	50/103 (48.5)	
Ever tested for hepatitis C			
No	203/977 (20.8)	38/108 (35.2)	**<0.001**
Yes	669/977 (68.5)	55/108 (50.9)	
Don’t know	105/977 (10.7)	15/108 (13.9)	
Tested for HCV in the past 2 years			
No	441/902 (48.9)	63/103 (61.2)	**0.022**
Yes	461/902 (51.1)	40/103 (38.8)	

*Asked only of self-reported HIV negative or unknown participants.

Multivariate logistic regression revealed that Asian ethnicity was independently associated with an increased odds of not having tested for HIV in the past 2 years (adjusted odds ratio [AOR] = 2.04; 95% confidence interval [CI]) 1.08-3.86) and hepatitis C (AOR = 2.30; 95% CI 1.27-4.19), but only for participants under the age of 35 (Tables 4a and b). Both models included adjustments for first language, age, and disclosure of male sex partners. In addition, the HIV testing model was adjusted for cigarette smoking and history of injection drug use. We did not find any associations between Asian ethnicity and ever testing for syphilis (Table 4c) or gonorrhea (data not shown) in either age group in adjusted models.

**Table 4:** Adjusted Confounder Model for not having tested for HIV, hepatitis C virus and syphilis in the past 2 years, stratified by age

**Variable**	**<35**	**p-value**	**≥35**	**p-value**
	**Adjusted odds ratio (95% CI)**		**Adjusted odds ratio (95% CI)**	
**a. Adjusted Confounder Model for not having tested for HIV in the past 2 years, stratified by age**				
Asian ethnicity	2.04 (1.08-3.86)	**0.028**	0.65 (0.28-1.51)	0.32
English or French as first language	1.29 (0.69-2.43)	0.426	1.28 (0.62-2.66)	0.51
Age (in years) of participate at study enrolment.	0.97 (0.92-1.02)	0.245	1.01 (0.98-1.03)	0.58
IDU ever	0.81 (0.41-1.56)	0.522	0.42 (0.20-0.86)	**0.017**
At the present time, do you smoke cigarettes?	1.31 (0.84-2.03)	0.234	1.15 (0.68-1.95)	0.60
Not told doctor of male sex partners	5.83 (3.67-9.24)	**<0.001**	3.47 (1.90-6.35)	**<0.001**
**b. Adjusted Confounder Model for not having tested for Hepatitis C Virus in the past 2 years, stratified by age**				
Asian ethnicity	2.30 (1.27-4.19)	0.006	1.05 (0.51-2.15)	0.90
English or French as first language	1.25 (0.71-2.22)	0.435	1.14 (0.61-2.13)	0.68
Age (in years) of participate at study enrolment.	0.99 (0.95-1.04)	0.664	1.02 (1.00-1.04)	0.063
Not told doctor of male sex partners	7.06 (4.30-11.60)	<0.001	2.43 (1.41-4.18)	0.001
**c. Adjusted Confounder Model for not having tested for syphilis in the past 2 years, stratified by age**				
Asian ethnicity	0.85 (0.45-1.61)	0.625	0.67 (0.32-1.40)	0.29
English or French as first language	1.19 (0.66-2.15)	0.563	0.81 (0.43-1.52)	0.51
Age (in years) of participate at study enrolment.	0.96 (0.92-1.01)	0.124	0.99 (0.97-1.01)	0.48
IDU ever	1.59 (0.86-2.93)	0.137	1.77 (1.08-2.90)	0.023
At the present time, do you smoke cigarettes?	0.66 (0.44-1.00)	0.048	0.65 (0.43-0.98)	0.038
Not told doctor of male sex partners	0.11 (0.07-0.18)	<0.001	0.24 (0.14-0.42)	<0.001

## Discussion

We found that approximately 10% of this sample of MSM from Vancouver self-identified as having Asian ancestry. While this represented the largest ethnic minority in our sample, this proportion is far below the estimated 40% of the population of Greater Vancouver who have Asian heritage [3]. This raises important questions regarding the underlying structure of the MSM population in Vancouver and how well venue- and event-based time-location sampling can appropriately sample this population. Other studies from large cities in North America have generally found that individuals of Asian ethnicity are underrepresented in studies of MSM [12-14]. However, it is worth noting that approximately 9.1% of the new positive HIV tests reported in men in BC over the 2003 – 2009 period were among men with Asian ethnicity [4], and this proportion appears to be increasing in recent years [15].

We found that HIV risk behaviour did not differ substantially between Asian and non-Asian MSM in our sample, except that Asian MSM were less likely to report more than five lifetime sexual partners, which may be partly a result of the younger age of Asian participants in this study. In particular, self-reported HIV negative or unknown sero-status Asian MSM reported unprotected anal intercourse with a known HIV positive or unknown serostatus partner at the same frequency as non-Asian MSM. Despite this, Asian participants in our study were less likely to have ever tested for both HIV and hepatitis C and this association remained for study participants under the age of 35 years, even after adjusting for other important determinants of testing such as having told a health care provider that they have male sex partners.

HIV prevalence, however, was found to be significantly lower among Asian MSM (3.7%) in our study compared to non-Asian MSM (19.0%), and ancillary risk factors for HIV transmission such as use of alcohol prior to sex and ever using injection drugs were reported at a lower frequency among Asian men in this sample. This may imply that the HIV risk environment for Asian MSM is different than for other MSM. It is also worth noting that while Asian MSM reported being less likely to attend gay bars, they were just as likely to look for sex partners in gay bars as non-Asian MSM.

Lower HIV testing rates in our sample may be due to a lack of awareness of local HIV testing services or guidelines [16,17]. However, we found that testing for gonorrhea or chlamydia was not significantly lower in Asian MSM, suggesting that perceptions around HIV risk may be different from risk for other STIs in this population. Previous studies have indicated that Asian immigrants were less likely to use health care services due to cultural and language barriers [17,18]. This is similar to studies in the United States where lower HIV testing rates are found amongst Asians and Pacific Islanders as a group [19,20].

Lower testing rates may also be due to less knowledge and less perceived risk for HIV and other STIs [17,21]. This suggests that more sexual health promotion services targeting younger Asian MSM should be implemented. In addition, disclosure to health care professionals about male sexual partners was independently and strongly associated with testing for HIV/STI. Therefore, any barriers within the healthcare setting which preclude a patient’s comfort in disclosing same sex sexual partners ought to be addressed to ensure proper care and timely testing. Healthcare providers may require further training in sensitivity and cultural competence to foster improved patient rapport. Cultural barriers such as homophobia in Asian societies [17] may play a crucial role, making Asian MSM less inclined to disclose information about same-sex sexual partners.

Our study revealed that nearly 40% of Asian MSM spoke a language other than English as their first language. This highlights the need to provide health care and prevention programs in languages other than English, especially in large metropolitan areas.

Readers should be cautious when interpreting our findings. The data presented here can be considered representative of the population of Asian MSM who attended the community venues sampled in the survey, and may not represent all Asian MSM in Vancouver. The venues we sampled may not have been ones that are most commonly frequented by Asian MSM. Furthermore, only those who were comfortable disclosing personal information and providing blood samples would have participated in this research study, thereby potentially excluding participants who may be even less likely to get tested. Because this survey was only administered in English, it may have limited participation of those who do not feel comfortable reading in English. As well, the relatively small proportion of individuals who self-identified as having Asian heritage may have limited our ability to demonstrate associations with some of the outcomes we observed. Finally, self-reported behavioural data may be subject to social desirability bias.

## Conclusions

We found a much smaller proportion of men who identified as being of Asian ethnicity in our sample of MSM in Vancouver than in the Vancouver population as a whole. We also found that the Asian MSM in this study had a lower prevalence of HIV in comparison to men of other ethnicities, but that the likelihood of having been tested for either hepatitis C or HIV was also lower for younger Asian MSM. This highlights the need for HIV prevention programs and further research which can more appropriately engage MSM of Asian and other ethnic minorities in Vancouver and elsewhere in North America.

## Competing interests

The authors declare that they have no competing interests.

## Authors’ contributions

TMM and MG developed the concept for the paper and planned the initial analysis. TMM wrote the first draft. BC and DMM revised the analysis plan and the early drafts of the paper. KC and SK conducted the analyses. WM, RH, WR, NM and RG all provided input into writing the manuscript. All authors approved the final version.

## Authors’ information

Members of the ManCount Study Team

Principal investigators

Mark Gilbert – BC Centre for Disease Control

Robert S. Hogg – BC Centre for Excellence in HIV/AIDS

Reka Gustafson – Vancouver Coastal Health

Chris P. Archibald-Public Health Agency of Canada

Tom Wong-Public Health Agency of Canada

Co-investigators

Community Based Research Centre

Dr. Terry Trussler

Dr. Rick Marchand

Health Initiative for Men

Phillip Banks

Wayne Robert

Jim Sheasgreen

Vancouver Coastal Health

Michael Kwag

Meaghan Thumath

Public health agency of Canada

Marissa McGuire

Susanna Ogunnaike-Cooke

Gayatri Jayaraman

Maureen Perrin

Stephanie Totten

Liz Venditti

BC Centre for excellence in HIV/AIDS

Dr. David Moore

Steve Kanters

Warren Michelow

Arn Schilder

University of British Columbia

Dr. Paul Gustafson

## Pre-publication history

The pre-publication history for this paper can be accessed here:

http://www.biomedcentral.com/1471-2458/13/763/prepub
